# Predictive Value of Adiponectin for Long-Term MACEs in Non-Diabetic STEMI Patients

**DOI:** 10.3390/jcm14227936

**Published:** 2025-11-09

**Authors:** Xhevdet Krasniqi, Josip Vincelj, Ibadete Bytyçi, Blerim Berisha, Aurora Bakalli

**Affiliations:** 1 Department of Internal Medicine, Medical Faculty, University of Prishtina “Hasan Prishtina”, 10000 Prishtina, Kosovo; xhevdet.krasniqi@uni-pr.edu (X.K.); ibadete.bytyci@uni-pr.edu (I.B.); aurora.bakalli@unipr.edu (A.B.); 2 Department of Cardiology, University Clinical Center of Kosova, 10000 Prishtina, Kosovo; 3 Department of Cardiovascular Medicine, Dubrava University Hospital, 10000 Zagreb, Croatia; jvinceljgg@gmail.com; 4 Department of Cardiology, Kreisklinik Roth, 91154 Roth, Germany; 5 Department of Medical Sciences, UBT-College, 10000 Prishtina, Kosovo

**Keywords:** adiponectin, MACE, STEMI

## Abstract

**Background:** A decreased level of adiponectin is known as a predictor of adverse left ventricular remodeling and major adverse cardiac events (MACEs). We evaluated long-term MACEs following ST-segment elevation myocardial infarction (STEMI) in relation to adiponectin levels. **Methods:** This prospective study included a total of 73 consecutive STEMI patients. Adiponectin, CK, CK-MB, cTnI, CRP, HDL cholesterol, LDL cholesterol, triglycerides, and other routine laboratory parameters were considered, and myocardial revascularization and two-dimensional echocardiography were performed. Subjects were divided into two groups according to their serum adiponectin concentrations. **Results:** In total, 24 (32.87%) patients suffered from MACEs, 19 (26.02%) with adiponectin value ≤ 1.8 ng/mL (group 1) and 5 (6.84%) with adiponectin value > 1.8 ng/mL (group 2) (*p* < 0.013). Heart failure (Killip >1) was present in 14 cases (19.17%) in group 1 and in 3 cases (4.1%) in group 2 (*p* < 0.001). Kaplan–Meier analysis was used to depict the occurrence of MACEs according to the adiponectin threshold identified during hospitalization (1.8 ng/mL). The log-rank test revealed a statistically significant difference in survival between groups (*p* = 0.013), and the AUC value for adiponectin was 0.77 (95% CI, 0.66–0.89), *p* = 0.01. Based on univariate logistic regression analysis, adiponectin and BMI were significantly associated with MACEs (*p* = 0.018, *p* = 0.034). Multivariate logistic regression analysis shows that serum adiponectin predicts MACEs after STEMI (*p* = 0.011). **Conclusions:** We found significant associations between adiponectin levels and MACEs in patients who survived STEMI. The established cut-off value of 1.8 ng/mL for adiponectin during hospitalization identified patients at risk for MACEs.

## 1. Introduction

Adiponectin, a 244-amino acid protein hormone secreted by adipose tissue, is encoded by the ADIPOQ gene localized to human chromosome 3q27. This novel adipokine was first identified in mice by Scherer et al., who termed it the “Adipocyte complement-related protein of 30 kDa” (Arcp30).

Matsuzawa and colleagues identified the human homologue in adipose tissue, referring to it as “adipose most 2 abundant gene transcript 1” (apM1). Tomita and colleagues identified adiponectin as a “gelatin-binding protein”, reporting its molecular mass to be nearly 28 kDa (GBP28) [1,2,3,4].

Recent studies have extended the sources of adiponectin beyond adipose tissue, demonstrating its synthesis and secretion by human cardiomyocytes [5]. Circulating adiponectin represents about 0.02% of the plasma protein and modulates a number of metabolic functions via receptors ADIPOR1, ADIPOR 2, and T-Cad [6,7,8]. Remarkably, Denzel et al. provided evidence that T-cadherin is necessary for adiponectin binding to cardiomyocytes [9,10].

Adiponectin, in association with other adipokines, has been shown to induce AMPK-Akt-phosphorylation and to potentiate anti-inflammatory peroxisome-proliferator-activated-receptor (PPAR)-a-effect [11,12]. Furthermore, adiponectin enhances nitric oxide (NO) both directly, via AMPK activation, and indirectly, by reducing the glucose level, contributing to vasodilatory cardioprotective effects and angiogenesis. Evidence suggests that PPAR agonists may serve as valuable therapeutic targets in the reduction of cardiovascular risk [13,14,15].

**Figure 1:**
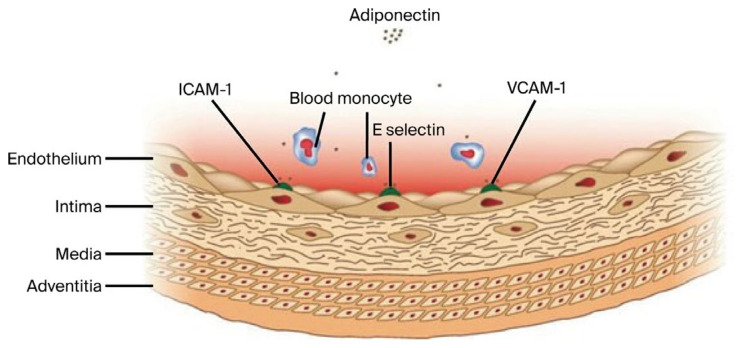
Inhibitory effects of adiponectin on the expression of adhesion molecules ICAM-1, VCAM-1, and E-Selectin.

Ouchi et al. were the first to describe the vascular effects of adiponectin on endothelial vascular cells, reporting its ability to inhibit the expression of adhesion molecules, including intracellular adhesion molecule-1 (ICAM-1), vascular cell adhesion molecule-1 (VCAM-1), and E selectin [16,17,18] (Figure 1). ICAM-1, as a proinflammatory mediator, is associated with elevated soluble suppression of tumorigenicity-2 (sST2) levels, facilitating the recruitment and activation of inflammatory cells within the injured vascular wall. Through inhibition of the interleukin-33/suppression of tumorigenicity-2 (IL-33/ST2) axis, which mediates group 2 innate lymphoid cells (ILC2) activation, adiponectin exerts anti-inflammatory effects counteracting the proinflammatory milieu reflected in elevated sST2 concentrations [19,20].

**Figure 2:**
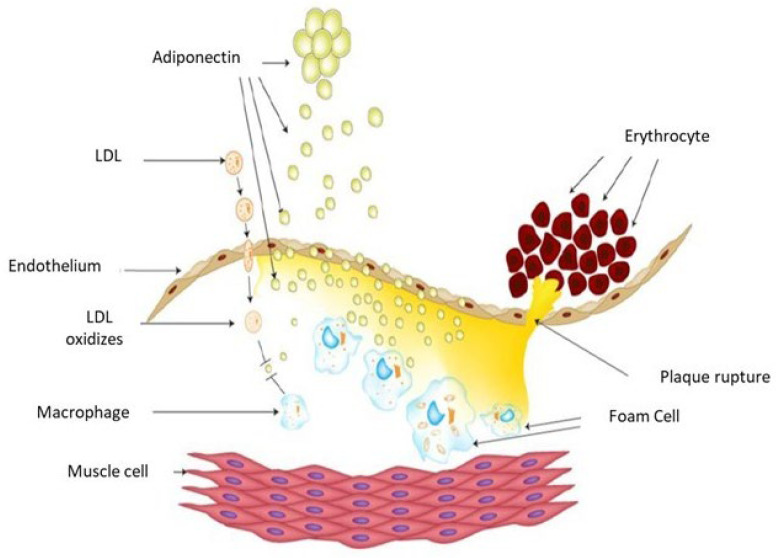
Schematic drawing of the atherosclerosis process. Adiponectin inhibits OxLDL and reduces macrophage transformation into foam cells, leading to its consumption in circulating plasma.

The anti-atherosclerotic effects of adiponectin are mediated through its anti-inflammatory actions and anti-atherogenic mechanisms [21,22,23,24] (Figure 2). Oxidative modification of low-density lipoprotein (LDL), involving both lipid and protein components, produces oxidized LDL (OxLDL). Elevated levels of OxLDL in the circulation and vascular wall contribute to endothelial dysfunction by downregulating eNOS expression, promoting HIF-1a accumulation, and inducing HIF-1-dependent gene activation in macrophages via the redox-mediated pathway [25,26,27,28,29,30].

Experimental studies demonstrate that adiponectin inhibits OxLDL-induced cell proliferation by suppressing cellular superoxide generation, thereby reducing the transformation of macrophages into foam cells [31,32]. Fibroblast growth factor 21 (FGF21) further protects against atherosclerosis by stimulating adiponectin expression in adipose tissue and inhibiting cholesterol synthesis in the liver [33,34,35].

Adiponectin protects against cardiac injury and failure by activating AMPK to prevent apoptosis and, via COX-2, suppressing TNF-ɑ production to reduce post-infarction systolic dysfunction [36,37,38,39,40,41].

Hemodynamic overload arising from increased vascular stiffness and peripheral resistance elevates left ventricular filling pressures in diastolic dysfunction and limits systolic reverse following myocardial infarction, which may contribute to heart failure and poor outcomes. Clinical evidence indicates that plasma adiponectin is an independent predictor of left ventricular systolic dysfunction, while reduced adiponectin levels are associated with diastolic dysfunction [42,43,44]. In an animal model of myocardial infarction, FGF21 gene transfer via intramuscular adenoviral injection improved systolic function after two weeks, an effect that was mediated through adiponectin signaling [45].

To our knowledge, no prior study has investigated the predictive value of adiponectin on long-term MACEs in STEMI patients.

The aim of this study is to evaluate the impact of hypoadiponectinemia as an independent predictor of major adverse cardiac events following STEMI.

## 2. Methods

### 2.1. Study Design

In this prospective study, 73 consecutive patients with ST-segment elevation myocardial infarction (STEMI) were included and divided into two groups according to their serum adiponectin concentrations (group 1 (adiponectin value ≤ 1.8 ng/mL) and group 2 (adiponectin value > 1.8 ng/mL)). Inclusion criteria for STEMI were as follows: (a) detection of cardiac biomarkers (cardiac troponin (cTn)) (ESC 0 h/1 h and 0 h/2 h algorithms), (b) symptoms of ischemia, and (c) ECG changes including new ST elevation at the J-point in at least two contiguous leads (≥2.5 mm in men <40 years, ≥2 mm in men ≥40 years, or ≥1.5 mm in women regardless of age in leads V2-V3 or ≥1 mm in the other leads), provided that left ventricular hypertrophy (LVH) or left bundle branch block (LBBB) were absent [46]. The exclusion criteria were previous myocardial infarction, previous diabetes mellitus, renal insufficiency, inflammatory disease, and acute infectious disease.

Clinical history was evaluated with regard to established coronary risk factors (dyslipidemia, arterial hypertension, and smoking), previous medications, and time from onset to admission. During the follow-up period, the risk of reinfarction appears to be especially driven by baseline characteristics and treatment patterns, rather than the infarct type itself (STEMI or NSTEMI) [47].

Laboratory parameters included adiponectin, creatine kinase (CK), the MB fraction of creatine kinase (CK-MB), cTnI, CRP, HDL cholesterol, LDL cholesterol, triglycerides, and routine parameters. Blood samples were obtained approximately 30 min after admission. Serum intended for adiponectin measurement was stored at −70 °C until biochemical analyses were performed. Serum adiponectin concentrations were measured with the ELISA method at room temperature (20–23 °C) in accordance with the manufacturer’s instructions using Phoenix Pharmaceuticals ELISA kits (Awareness Technologies Inc: UCCK, Prishtina), Kosovo; and a Siemens BEP 2000 (Dubrava University Hospital, Zagreb, Croatia).

Two-dimensional echocardiography was performed for all patients. The LV ejection fraction was assessed using the modified Simpson’s biplane method from apical 4- and 2-chamber views. Diastolic function was evaluated using mitral inflow PW Doppler (E/A, E wave deceleration time), tissue Doppler (E/e′), and pulmonary inflow PW Doppler (S, D) measurements and graded 0–3. Adverse LV remodeling was defined as a > 20% increase in LV end-diastolic or end-systolic volumes, while adverse diastolic remodeling was defined as ≥ 1 grade or persistence of a restrictive pattern at follow-up.

All patients underwent revascularization with periprocedural pharmacotherapy according to current guidelines. Post-PCI standard therapy included aspirin (100 mg), clopidogrel (75 mg), or prasugrel (10 mg); beta-blockers; lipid-lowering agents; and angiotensin-converting enzyme (ACE) inhibitors or angiotensin II receptor blockers (ARB), in line with international recommendations [46,48].

Over a follow-up period of one year, patients were prospectively evaluated for major adverse cardiac events (MACEs), comprising angina pectoris, reinfarction, congestive heart failure, stroke, and cardiac death.

This study was conducted in accordance with the Declaration of Helsinki and was approved by the institutional Ethics Committees of Dubrava University Hospital, Zagreb, and the University Clinical Center of Kosovo, Prishtina.

### 2.2. Statistical Analysis

Descriptive data are presented as means ± standard deviation (SD) for normally distributed variables and as median with interquartile range (IQR) for non-normally distributed variables. Comparisons between the groups were performed using Student’s *t* test, the Kruskal–Wallis test, the Mann–Whitney test, and the chi-square test, depending on the distribution and types of variables. Receiver operating characteristic (ROC) curve analysis provided a graphical representation of the true positive rate versus the false positive rate across different cut-off points, facilitating the selection of the optimal threshold for a given context. The area under the curve (AUC) quantified the predictive accuracy for binary outcomes. Univariate and multivariate logistic regression analyses were subsequently performed to identify predictors of major adverse cardiac events (MACEs). Kaplan–Meier estimates were used to assess the association between adiponectin levels and MACEs over a 12-month follow-up period. Kaplan–Meier survival analysis was applied since only one predictor was used (adiponectin).

MACEs were categorized as MACE type 1 (angina pectoris), 2 (myocardial reinfarction), 3 (heart failure), 4 (stroke), or 5 (death) for the purpose of statistical analysis. The cut-off of adiponectin was determined using the value of adiponectin as a test variable, and MACE type 3 was used as a state variable, resulting in an adiponectin value of 1.8 ng/mL. The log-rank test was used to compare the survival distributions of two samples. A two-tailed *p*-value of <0.05 was considered statistically significant. Data were analyzed using SPSS statistical software, version 26.

## 3. Results

### 3.1. Patient Characteristics

**Figure 3:**
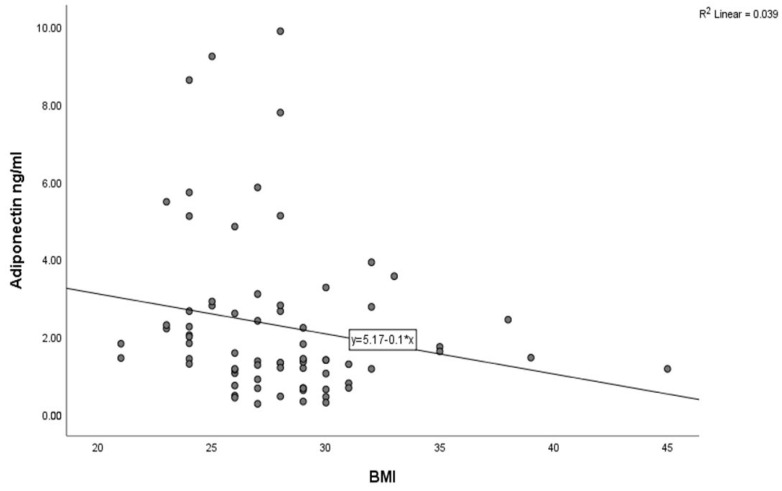
Correlation between adiponectin levels and BMI in the study population (Spearman’s correlation coefficient r = −0.269, *p* < 0.02).

**Figure 4:**
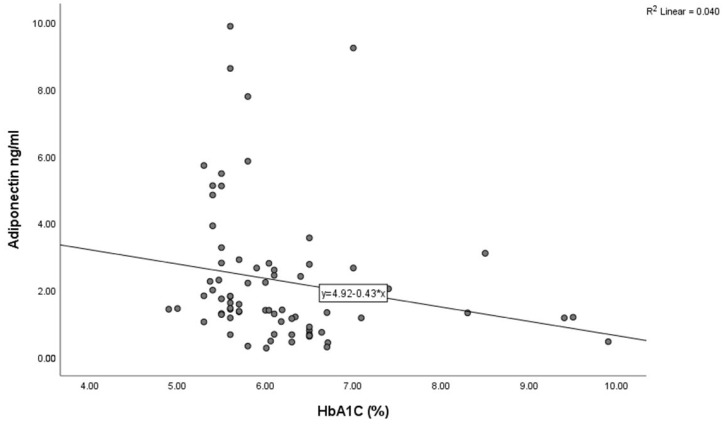
Correlation between adiponectin levels and HbA1C in the study population (Spearman’s correlation coefficient r = −0.384, *p* < 0.01).

The baseline characteristics of the study population are detailed in Table 1. The mean age was 59.91 ±11.21 years, and 55 (75.34%) participants were male. A total of 43 (58.9%), 11 (15.06%), and 37 (50.68%) patients had a medical history of hypertension, prediabetes mellitus, and smoking, respectively. In terms of the coronary angiographic findings, the number of patients with multivessel coronary artery disease was 22 (30.13%). An inverse correlation between adiponectin and BMI was observed using Spearman’s correlation analysis (Figure 3, *p* = 0.02) and HbA1C (Figure 4, *p* < 0.001) in all study subjects. The comparison of body mass index (BMI) and HbA1C between groups with different levels of adiponectin (≤1.8 ng/mL and >1.8 ng/L) was statistically significant (the *p*-value was 0.003 for BMI and 0.023 for HbA1C).

### 3.2. Risk Factors and MACEs

To identify factors associated with the occurrence of MACEs, we performed univariate logistic regression analysis including age, gender, body mass index, waist circumference, systolic blood pressure, diastolic blood pressure, adiponectin, LDL cholesterol, HDL cholesterol, triglycerides, fasting glucose, creatinine, and CRP levels. Adiponectin and BMI were significantly associated with MACEs (*p* = 0.018, *p* = 0.034) (Table 2). In multivariate logistic regression analysis, serum adiponectin independently predicted the occurrence of MACEs after STEMI (*p* = 0.011) (Table 3).

### 3.3. Adiponectin and MACEs

**Figure 5:**
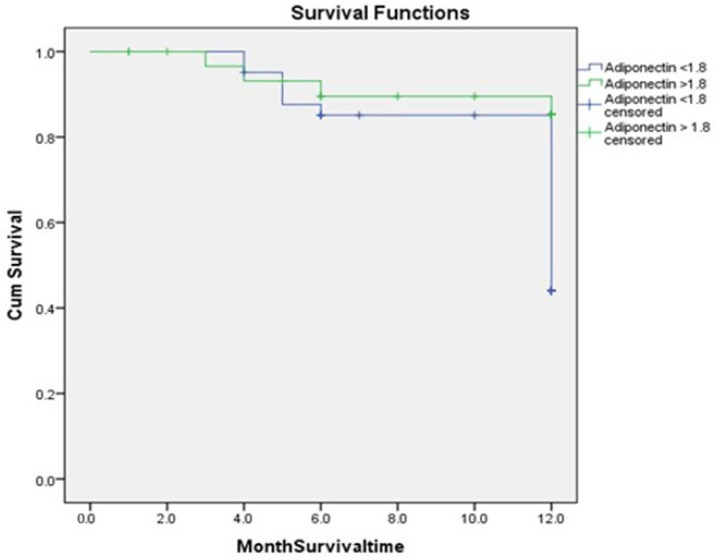
Kaplan–Meier estimates showing higher recurrence rates of MACEs among patients with adiponectin levels ≤ 1.8 compared to higher adiponectin levels > 1.8 (*p* = 0.013).

In total, 24 patients (32.87%) experienced MACEs, 19 (26.02%) with adiponectin values ≤ 1.8 ng/mL and 5 (6.84%) with values > 1.8 ng/mL (*p* < 0.013) (Table 1). Heart failure (Killip > 1) occurred in 14 cases (19.17%) in group 1 and in 3 cases (4.1%) in group 2 (*p* < 0.001). Kaplan–Meier curves were used to show the number of MACEs and the proportion of patients that survived at each even time point based on the cut-off value of adiponectin during hospitalization (1.8 ng/mL) (Figure 5) [49]. The log-rank test demonstrated a significant difference in survival between groups (*p* = 0.013).

**Figure 6:**
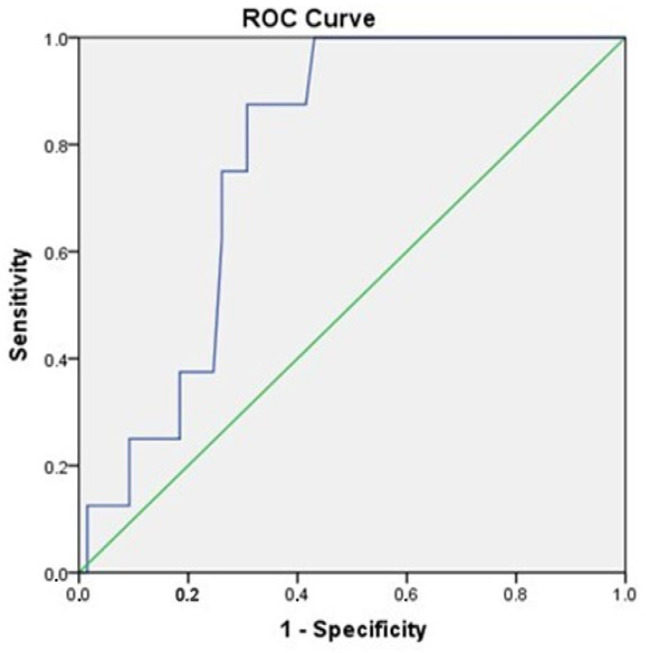
ROC curve analysis of adiponectin for the prediction of MACEs (95% CI, 0.66–0.89), *p* = 0.01.

A receiver operating characteristic (ROC) curve, which plots the true positive rate against the false positive rate across varying cut-off points, showed an AUC of 0.77 (95% CI, 0.66–0.89), *p* = 0.01 (Figure 6) [49].

Table 4 presents the area under the curve (AUC) values for the biomarkers (troponin I, creatine kinase, creatine kinase-MB, adiponectin, and C-reactive protein).

## 4. Discussion

In this study, we investigated the influence of adiponectin on the prediction of long-term MACEs in non-diabetic STEMI patients.

Collateral blood flow plays a crucial role after acute coronary ischemia. In our study, 30.12% of patients presented with multiple-vessel disease, with no significant difference between groups. Furthermore, low adiponectin levels were found in all patients, independent of anatomical localization and the number of diseased arteries. These findings imply that adiponectin may be involved in the pathophysiological response during the acute phase of coronary artery disease. One possible explanation is that the process of new microvessel formation after acute myocardial infarction may be associated with decreased circulating adiponectin concentrations. Consistent with this, recent evidence shows that adiponectin facilitates the migratory activity of endothelial progenitor cells [50,51]. Therefore, adiponectin may play a contributory role in revascularization processes, leading to its utilization and subsequent reduction in circulating plasma levels. Low plasma adiponectin levels may also relate to microvascular myocardial ischemia or impaired myocardial energy utilization [52,53]. We speculate that cellular-level consumption of endogenous adiponectin during neovascularization after acute coronary syndrome may lead to hyperglycemia. In our study, HbA1c values differed significantly between the two adiponectin groups (*p* = 0.023). These findings suggest that the degree of hyperglycemia reflects the severity of hypoadiponectinemia, which in turn may activate counter-protective mechanisms responsible for major adverse cardiac events.

In our study, the participants were not within the normal or healthy weight range, and a significant difference was observed between the two groups. Previous research reported that weight reduction therapy increased the plasma adiponectin concentration, and an inverse correlation of adiponectin levels with body weight and obesity has been well established [54,55,56]. The present findings confirm this association in non-diabetic patients.

Our findings confirmed that adiponectin inversely correlated with HbA1C and BMI (Figure 3 and Figure 4), while all of these parameters were associated with MACEs, supporting the idea that adiponectin alone or together with HbA1C level and not just HbA1C alone predicts the cardiovascular risk. In non-diabetic STEMI patients, adiponectin appears to reflect not only chronic metabolic risk but also acute insulin-resistance states and endothelial dysfunction that influence reperfusion efficacy and post-infarction remodeling. A lower preprocedural adiponectin level is strongly associated with increased insulin resistance, as reflected by higher HOMA-IR scores, and an increased risk of restenosis and adverse ischemic events, suggesting that hypoadiponectinemia identifies a metabolically vulnerable phenotype even in the absence of diagnosed diabetes [57]. Recent reports further indicate that adiponectin, induced via fibroblast growth factor 21, contributes to the regulation of energy and vascular homeostasis and provides protection against cardiometabolic disorders [34,35].

The present study demonstrated that serum adiponectin levels were indirectly associated with plasma HDL cholesterol concentrations, suggesting that HDL may serve as an intermediate factor in the relationship between serum adiponectin and coronary artery disease. Adiponectin was shown to increase the level of HDL through the inhibition of hepatic lipase expression. Both adiponectin and HDL have also been reported to increase nitric oxide production via activation of the AMPK-Akt-eNOS phosphorylation pathway, thereby contributing to vasodilatory cardioprotective effects [58,59,60,61,62]. Also, the combined action of statins (HMG-CoA reductase inhibitors) and adiponectin has been reported to upregulate eNOS expression [63,64]. Thus, the observed association between adiponectin and HDL may indirectly support the hypothesis that impaired angiogenesis and altered vascular tone regulation in non-diabetic patients following acute myocardial infarction play a pivotal role in the pathophysiology of major adverse cardiac events.

Adiponectin is closely related to metabolic regulation and is considered to exert both anti-atherogenic and anti-inflammatory effects. Previous studies have demonstrated an association between adiponectin and subclinical atherosclerosis [65,66]. In patients with significant coronary stenosis, coronary flow reserve has been correlated with vulnerable plaques and elevated CRP levels. However, in our study, no association was found between CRP levels and the prediction of MACEs, suggesting that CRP predominantly reflects the acute phase of coronary artery disease. We therefore hypothesize that, following acute coronary syndrome, patients may lose the protective effects of adiponectin, potentially due to its involvement in OxLDL inhibition and the consequent reduction in macrophage transformation into foam cells (Figure 2). This results in adiponectin consumption in the circulating plasma and a high level of CRP. Such a mechanism may explain both the consumption of adiponectin in the circulation and the concomitant elevation in CRP.

In STEMI patients, adiponectin and NT-proBNP have emerged as valuable biomarkers for predicting major adverse cardiac events (MACEs). Low adiponectin levels in the acute phase of myocardial infarction are associated with elevated left ventricular filling pressures due to diastolic dysfunction, adverse left ventricular remodeling, and increased MACE incidence. Conversely, elevated NT-proBNP reflects myocardial wall stress, neurohormonal activation, and impaired ventricular function, serving as an independent predictor of heart failure and mortality [67,68,69,70,71]. In our study, low levels of adiponectin in the acute phase were associated with high rates of MACEs after STEMI, with significant variability in admission-day adiponectin values observed among patients stratified by MACE occurrence [72]. A cut-off value of 1.8 ng/mL for adiponectin levels on the day of admission was set to perform survival analysis and examine the number of MACEs and corresponding survival rates.

Establishing biomarkers and their respective cut-offs for forecasting LV remodeling and long-term MACEs post-myocardial infarction provides valuable insights into clinical management [73]. This approach enables early initiation of appropriate treatment for these patients.

One limitation is that this observational study had a relatively limited number of patients.

## 5. Conclusions

We found significant associations between adiponectin levels and MACEs in patients who survived acute ST-elevation myocardial infarction. The established cut-off value of 1.8 ng/mL for adiponectin during hospitalization identified patients at risk for MACEs.

## Figures and Tables

**Table 1:** Baseline characteristics of patients.

Characteristics	Adiponectin ≤ 1.8 ng/mL (n = 41)	Adiponectin > 1.8 ng/mL (n = 32)	*p*-Value
Age, years	59.68 (±10.68)	60.14 (±11.75)	0.78
Gender, n (%)	20 (27.39)	25 (34.24)	0.42
BMI (kg/m^2^)	26.66 (3.57)	29.00 (4.05)	0.003
Hypertension, n (%)	18 (24.65)	25 (34.24)	0.99
Smoking, n (%)	16 (21.91)	21 (28.76)	0.91
Ejection fraction, %	51.59 (±9.19)	53.34 (±8.69)	0.64
Adverse diastolic remodeling (Diastolic function grade ≥ 1, n (%)	25 (34.24)	28 (38.35)	<0.001
Heart failure (Killip > 1), n (%)	14 (19.17)	3 (4.1)	<0.001
MACEs, n (%)	19 (26.02)	5 (6.84)	0.013
Hemoglobin (mg/dL)	137.68 (±14.82)	135.61 (±14.41)	0.80
Creatine kinase (U/I)	1502.0 (42.0–7550.0)	1298 (245.0–4764.0)	0.94
Creatine kinase-MB (U/I)	161.0 (15.0–929.0)	152.0 (19.5–500.0)	0.88
Troponin I (ng/mL)	45.01 (0.01–180.0)	5.46 (0.01–137.0)	0.03
C-reactive protein (mg/L)	8.49 (±13.84)	25.68(±54.47)	0.17
LDL cholesterol (mmol/L)	3.34 (±1.08)	3.68 (±0.82)	0.063
HDL cholesterol (mmol/L)	1.01 (±0.32)	1.17 (±0.40)	0.043
Multivessel coronary artery disease, n (%)	7 (9.58)	15 (20.54)	0.54
HbA1C, %	6.34 (±1.10)	5.93(±0.71)	0.023
Final TIMI grade flow ≤ 2, n (%)	7 (9.58)	5 (6.84)	0.15

BMI: body mass index; MACEs: major adverse cardiovascular events; LDL: low-density lipoprotein; HDL: high-density lipoprotein; HbA1C: glycated hemoglobin; TIMI: thrombolysis in myocardial infarction.

**Table 2:** Predictors of MACEs in univariate regression analysis.

Parameter	OR	95% CI	*p*-Value
Age, years	1.005	0.125–1.124	0.81
Gender (male)	0.375	0.000–12.44	0.08
BMI (kg/m^2^)	1.161	1.011–1.333	0.034
Waist circumference (cm)	1.051	1.0–1.106	0.51
Systolic BP (mmHg)	0.966	0.973–1.020	0.733
Diastolic BP (mmHg)	0.990	0.951–1.030	0.615
Adiponectin (ng/mL)	0.531	0.314–0.896	0.018
HbA1C, %	2.062	1.132–3.757	0.018
LDL (mmol/L)	1.125	0.683–1.853	0.643
HDL (mmol/L)	0.903	0.235–3.465	0.882
CRP (mg/L)	1.022	0.999–1.046	0.058
Triglyceride (mmol/L)	0.760	0.772–1.425	0.760
Fasting glucose (mmol/L)	1.351	1.051–1.736	0.019
Urea (mmol/L)	1.062	0.894–1.262	0.491
Creatinine (μmol/L)	1.0	0.981–1.020	0.998

OR: odds ratio; CI: confidence interval; BMI: body mass index; BP: blood pressure; HbA1C: glycated hemoglobin; HDL: high-density lipoprotein; LDL: low-density lipoprotein.

**Table 3:** Multivariate regression analysis for MACEs.

MACE	OR	95% CI	*p*-Value
Adiponectin (ng/mL)	2.964	1.27–6.86	0.011
HbA1C, %	0.687	0.326–1.44	0.324
HDL (mmol/L)	0.105	0.011–0.961	0.046
LDL (mmol/L)	0.68	0.289–1.599	0.376
BMI (kg/m^2^)	0.886	0.732–1.072	0.214
Systolic BP (mmHg)	1.011	0.969–1.056	0.605
LVEF%	0.946	0.842–1.062	0.345
Heart failure (Killip class > 1)	0.88	0.008–0.97	0.047
Multiple vessel > 1	0.103	0.014–0.719	0.022

OR: odds ratio; CI: confidence interval; BMI: body mass index; BP: blood pressure; HbA1C: glycated hemoglobin; HDL: high-density lipoprotein; LDL: low-density lipoprotein; LVEF: left ventricular ejection fraction.

**Table 4:** Area under the curve values for biomarkers.

Biomarker	AUC (95% CI)	Cut-Off Value	*p*-Value
Adiponectin (ng/mL)	0.77 (0.66–0.89)	1.80	0.01
HbA1C, %	0.74 (0.54–0.95)	6.35	0.02
Troponin I (ng/mL)	0.60 (0.39–0.81)	31.75	0.32
Creatine kinase (U/I	0.60 (0.44–0.77)	1405	0.32
Creatine kinase-MB (U/I)	0.51 (0.33–0.69)	169.5	0.90
Hemoglobin (mg/dL)	0.60 (0.41–0.79)	140.5	0.32
C-reactive protein (mg/L)	0.78 (0.59–0.98)	7.0	0.008

HbA1C: glycated hemoglobin.

## Data Availability

The data presented in this study are available from the corresponding author upon reasonable request due to privacy and ethical considerations.
